# Thyroid Cancer Risk Is Not Increased in Diabetic Patients

**DOI:** 10.1371/journal.pone.0053096

**Published:** 2012-12-27

**Authors:** Chin-Hsiao Tseng

**Affiliations:** 1 Department of Internal Medicine, National Taiwan University College of Medicine, Taipei, Taiwan; 2 Division of Endocrinology and Metabolism, Department of Internal Medicine, National Taiwan University Hospital, Taipei, Taiwan; 3 Division of Environmental Health and Occupational Medicine of the National Health Research Institutes, Taipei, Taiwan; Consiglio Nazionale delle Ricerche (CNR), Italy

## Abstract

**Objective:**

This study evaluated thyroid cancer risk with regards to diabetes status and diabetes duration, and with the use of anti-diabetic drugs including sulfonylurea, metformin, insulin, acarbose, pioglitazone and rosiglitazone, by using a population-based reimbursement database in Taiwan.

**Methods:**

A random sample of 1,000,000 subjects covered by the National Health Insurance was recruited. After excluding patients with type 1 diabetes, 999730 subjects (495673 men and 504057 women) were recruited into the analyses. Logistic regression estimated the odds ratios (OR) and their 95% confidence intervals (CI) for independent variables including age, sex, diabetes status/duration, anti-diabetic drugs, other medications, comorbidities, living regions, occupation and examinations that might potentially lead to the diagnosis of thyroid cancer in various models.

**Results:**

The diabetic patients had a significantly higher probability of receiving potential detection examinations (6.38% vs. 5.83%, *P*<0.0001). After multivariable-adjustment, the OR (95% CI) for diabetes status was 0.816 (0.652–1.021); and for diabetes duration <1 year, 1–3 years, 3–5 years and ≥5 years vs. non-diabetes was 0.071 (0.010–0.507), 0.450 (0.250–0.813), 0.374 (0.203–0.689) and 1.159 (0.914–1.470), respectively. Among the anti-diabetic agents, only sulfonylurea was significantly associated with thyroid cancer, OR (95% CI): 1.882 (1.202–2.947). The OR (95% CI) for insulin, metformin, acarbose, pioglitazone and rosiglitazone was 1.701 (0.860–3.364), 0.696 (0.419–1.155), 0.581 (0.202–1.674), 0.522 (0.069–3.926) and 0.669 (0.230–1.948), respectively. Furthermore, patients with benign thyroid disease or other cancer, living in Kao-Ping/Eastern regions, or receiving potential detection examinations might have a significantly higher risk; and male sex, hypertension, dyslipidemia, chronic obstructive pulmonary disease, vascular complications or use of statin, aspirin or non-steroidal anti-inflammatory drugs might be associated with a significantly lower risk.

**Conclusions:**

There is a lack of an overall association between diabetes and thyroid cancer, but patients with diabetes duration <5 years have a significantly lower risk. Sulfonylurea may increase the risk of thyroid cancer.

## Introduction

The incidence of thyroid cancer is increasing worldwide [1]–[4]. In the USA it increased by 2.4-fold from 1973 to 2002 [1]; and in Taiwan it increased from an age-standardized rate of 1.44 per 100,000 in 1980–1984 to 5.68 per 100,000 in 2000–2006 [2]. Women have a higher incidence of thyroid cancer than their male counterparts in different ethnicities [2], [3]. Although such an increasing trend can be partially explained by the enhanced detection of early-stage tumors by the use of thyroid ultrasound and ultrasound-guided fine needle aspiration cytology examination, these cannot explain the increasing rates preceding the widespread use of the ultrasound and the increased incidence of large (>5 cm) papillary thyroid cancer [5]. Environmental exposure to ionizing radiation and benign thyroid conditions are well recognized risk factors for thyroid cancer and may also contribute to such an increasing incidence [3].

Patients with type 2 diabetes mellitus have an increased risk of cancer involving liver, colon, endometrium, pancreas, urinary bladder and breast [6], [7]. However, whether diabetic patients may have a higher risk of thyroid cancer is rarely studied and the findings from different studies are inconclusive [5]. Several features of diabetes may potentially affect the mitogenic pathways of the follicular cells and may increase the risk of thyroid cancer in the diabetic patients [5]. The proposed mechanisms for such a potential link include insulin resistance, increased insulin level, increased glucose or triglycerides, obesity and increased level of thyroid-stimulating hormone (TSH) [5].

A US prospective study observed a non-significantly higher risk of total thyroid cancer associated with diabetes, hazard ratio (95% confidence interval): 1.25 (0.95–1.64) [8]. A recent meta-analysis including 5 US-based prospective cohort studies concluded that self-reported diabetes has no overall association with thyroid cancer, with hazard ratio (95% confidence interval): 1.08 (0.83–1.40) [9]. An Israeli retrospective cohort study also did not show a significant association between diabetes and thyroid cancer in either men or women [10]. However, another 12-year prospective follow-up study including seven population-based cohorts in Norway, Austria and Sweden concluded that there was an inverse association between blood glucose and thyroid cancer risk in women, but not in men [11].

Studies conducted in the Asian populations are still scarce and results are also controversial. A hospital-based case-control study from China suggested that higher fasting plasma glucose and diabetes history are both significantly associated with a higher risk of thyroid cancer [12]. However, a hospital-based study in Taiwan contradictorily showed that diabetes was associated with a significantly lower risk of thyroid cancer [13]. To our knowledge, there has not been any population-based study conducted in the Asian populations and the association between anti-diabetic drugs and thyroid cancer has not been extensively investigated.

By using the population-based reimbursement database of the National Health Insurance (NHI) of Taiwan, the aims of the present study were to evaluate 1) whether diabetic patients might have an increased risk of thyroid cancer in terms of diabetes status and diabetes duration; and 2) whether the anti-diabetic drugs, including insulin, sulfonylurea, metformin, acarbose, pioglitazone and rosiglitazone, might be associated with thyroid cancer.

## Materials and Methods

A single-payer NHI program was launched on March 1, 1995 in Taiwan. According to the Ministry of the Interior of Taiwan, in 2005, more than 98.0% of the Taiwanese population was covered by the NHI [7]. Each year, the Bureau of NHI collects data, including registration files and original claims data for reimbursement, and sends them to the National Health Research Institutes, the only institutes approved, as per local regulations, for managing academic research databases. The data files are then de-identified by scrambling the identification codes of patients and medical facilities for the protection of privacy. From January 1, 2005 to January 1, 2006, there were approximately 25.68 million beneficiaries in the NHI program according to the Registry for Beneficiaries data files. The National Health Research Institutes randomly sampled 1,000,000 beneficiaries from this registry file and created the Longitudinal Health Insurance Database 2005 (LHID 2005) by compiling all of the reimbursement data files for these sampled individuals for academic research [7]. The random sample was representative of the whole population [7]. The LHID 2005 was approved for use in this study, and the database contained all longitudinal reimbursement information of the random sample from 1996 to the end of 2005. Sex, birth date, medications, and diagnostic codes based on the International Classification of Diseases, Ninth Revision, Clinical Modification (ICD-9-CM) were retrieved for analyses. Diabetes was coded 250.1–250.9, and thyroid cancer 193. To assure the correct temporal sequence between cause and effect (thyroid cancer), diabetes status/duration, comorbidities, examinations and medications were counted only as they appeared before thyroid cancer in patients having a diagnosis of thyroid cancer.

After excluding patients with type 1 diabetes (in Taiwan, patients with type 1 diabetes were issued a “Severe Morbidity Card” after certified diagnosis), a total of 999730 subjects (495673 men and 504057 women) were recruited into the analyses.

To compare whether diabetic patients had a higher probability of receiving examinations that might potentially lead to the diagnosis of thyroid cancer than non-diabetic subjects, the following examinations were analyzed by Chi-square test: 1) thyroid sonography; 2) thyroid aspiration; 3) thyroid function test (T3, T4 and TSH); and 4) any of the above (considered as “potential detection examinations” in the following analyses).

Logistic regression models were created with diabetes being considered either as diabetes status (yes versus no: “diabetes status model”) or diabetes duration (non-diabetes, <1, 1–3, 3–5 and ≥5 years: “diabetes duration model”). Thyroid cancer was the dependent variable and the models were adjusted for age, sex, comorbidities, medications, living region, occupation and potential detection examinations. The comorbidities (ICD-9-CM codes) included hypertension (401–405), chronic obstructive pulmonary disease (490–496, a surrogate for smoking), stroke (430–438), nephropathy (580–589), ischemic heart disease (410–414), peripheral arterial disease (250.7, 785.4, 443.81, 440–448), eye disease (250.5, 362.0, 369, 366.41, 365.44), obesity (278), dyslipidemia (272.0–272.4), benign thyroid disease (240–246) and other cancer (140–208, excluding 193). Medications included in the full models were statin, fibrate, angiotensin-converting enzyme inhibitors and/or angiotensin receptor blockers, calcium channel blockers, sulfonylurea, metformin, insulin, acarbose, pioglitazone, rosiglitazone, aspirin, ticlopidine, clopidogrel, dipyridamole, and non-steroidal anti-inflammatory drugs (other than aspirin). Occupation was categorized as I: civil servants, teachers, employees of governmental or private business, professionals and technicians; II: people without particular employers, self-employed or seamen, III: farmers or fishermen; and IV: low-income families supported by social welfare or veterans. Living region was categorized as Taipei, Northern, Central, Southern and Kao-Ping/Eastern.

Analyses were conducted using SAS statistical software, version 9.1 (SAS Institute, Cary, NC). Data were expressed as mean (standard deviation) for continuous variables or number (%) for categorical variables. *P*<0.05 was considered as statistically significant.

## Results

Table 1 compares the frequency of examinations that might potentially lead to the diagnosis of thyroid cancer between diabetic and non-diabetic subjects. It is evident that the diabetic patients had a significantly higher probability of receiving these examinations.

**Table 1:** Comparisons of examinations potentially leading to diagnosis of thyroid cancer between patients with and without diabetes mellitus.

	Diabetes mellitus				
Examination	No		Yes		*P* value
	n	%	n	%	
	883802		115928		
**Thyroid sonography**					
No	880044	99.57	114805	99.03	<0.0001
Yes	3758	0.43	1123	0.97	
**Thyroid aspiration**					
No	880957	99.68	114904	99.12	0.0007
Yes	2845	0.32	1024	0.88	
**Thyroid function test**					
No	833176	94.27	97210	83.85	<0.0001
Yes	50626	5.73	18718	16.15	
**Potential detection examinations (any of the above)**					
No	832268	94.17	96944	83.62	<0.0001
Yes	51534	5.83	18984	6.38	

Table 2 shows the adjusted odds ratios for all covariates derived from the “diabetes status model”. Diabetes status was not significantly associated with thyroid cancer. Age and women consistently showed an increased risk. With regards to comorbidities, hypertension, dyslipidemia, chronic obstructive pulmonary disease, stroke, nephropathy, ischemic heart disease, peripheral arterial disease and eye disease were all significantly associated with a lower risk; while benign thyroid disease and other cancer showed a significantly higher risk. For the medications, statin, aspirin and non-steroidal anti-inflammatory drugs were significantly associated with a lower risk, but sulfonylurea showed a significantly higher risk. People living in the Kao-Ping/Eastern regions and patients receiving potential detection examinations had significantly higher risk.

**Table 2:** Adjusted odds ratios for thyroid cancer in diabetes status model.

Variables	Interpretation	Thyroid cancer case number	OR	95% CI	*P* value
***Basic characteristics***					
Age	Every 1-year increment	943	1.057	(1.053, 1.062)	<0.0001
Sex	Men vs. Women	186/757	0.424	(0.358, 0.503)	<0.0001
Diabetes status	Yes vs. No	149/794	0.816	(0.652, 1.021)	0.0756
Living region	Northern vs. Taipei	105/367	0.855	(0.686, 1.066)	0.1632
	Central vs. Taipei	162/367	0.951	(0.786, 1.150)	0.6017
	Southern vs. Taipei	123/367	0.991	(0.800, 1.227)	0.9324
	Kao-Ping/Eastern vs. Taipei	186/367	1.203	(1.002, 1.444)	0.0475
Occupation*	II vs. I	197/445	1.146	(0.965, 1.360)	0.1199
	III vs. I	143/445	0.917	(0.744, 1.131)	0.4187
	IV vs. I	158/445	0.855	(0.707, 1.033)	0.1037
Potential detection examinations	Yes vs. No	446/497	1.313	(1.082, 1.594)	0.0058
***Comorbidities***					
Hypertension	Yes vs. No	181/762	0.612	(0.479, 0.781)	<0.0001
COPD	Yes vs. No	147/796	0.432	(0.359, 0.520)	<0.0001
Stroke	Yes vs. No	52/891	0.627	(0.462, 0.851)	0.0027
Nephropathy	Yes vs. No	50/893	0.619	(0.460, 0.833)	0.0015
Ischemic heart disease	Yes vs. No	81/862	0.506	(0.389, 0.659)	<0.0001
Peripheral arterial disease	Yes vs. No	34/909	0.675	(0.473, 0.962)	0.0298
Eye disease	Yes vs. No	6/937	0.406	(0.174, 0.945)	0.0366
Obesity	Yes vs. No	7/936	0.577	(0.273, 1.221)	0.1504
Dyslipidemia	Yes vs. No	97/846	0.463	(0.361, 0.593)	<0.0001
Benign thyroid disease	Yes vs. No	550/393	23.094	(18.868, 28.267)	<0.0001
Other cancer	Yes vs. No	97/846	1.608	(1.291, 2.002)	<0.0001
***Anti-diabetic drugs***					
Sulfonylurea	Yes vs. No	52/891	1.882	(1.202, 2.947)	0.0057
Metformin	Yes vs. No	37/906	0.696	(0.419, 1.155)	0.1606
Insulin	Yes vs. No	11/932	1.701	(0.860, 3.364)	0.1268
Acarbose	Yes vs. No	4/939	0.581	(0.202, 1.674)	0.3146
Pioglitazone	Yes vs. No	1/942	0.522	(0.069, 3.926)	0.5276
Rosiglitazone	Yes vs. No	4/939	0.669	(0.230, 1.948)	0.4613
***Other medications***					
Statin	Yes vs. No	28/915	0.552	(0.360, 0.844)	0.0061
Fibrate	Yes vs. No	39/904	1.376	(0.957, 1.976)	0.0847
ACEI/ARB	Yes vs. No	115/828	0.886	(0.675, 1.163)	0.3825
Calcium channel blocker	Yes vs. No	158/785	1.255	(0.990, 1.592)	0.0609
Aspirin	Yes vs. No	138/805	0.767	(0.627, 0.939)	0.0101
Ticlopidine	Yes vs. No	7/936	1.242	(0.572, 2.697)	0.5843
Clopidogrel	Yes vs. No	1/942	0.197	(0.027, 1.428)	0.1079
Dipyridamole	Yes vs. No	122/821	0.974	(0.780, 1.217)	0.8171
NSAID	Yes vs. No	827/116	0.102	(0.082, 0.127)	<0.0001

* Categories of occupation are explained in Materials and Methods. OR: odds ratio; CI: confidence interval; COPD: chronic obstructive pulmonary disease; ACEI/ARB: angiotensin converting enzyme inhibitor/angiotensin receptor blocker; NSAID: non-steroidal anti-inflammatory drugs, other than aspirin.

Table 3 shows the adjusted odds ratios for the various diabetes durations in the “diabetes duration model”. Patients with a diabetes duration <5 years showed significantly lower risk of thyroid cancer and those having a diabetes duration ≥5 years showed a neutral association. The adjusted odds ratios for the other independent variables in this “diabetes duration model” were not shown in the table because they did not differ remarkably from their corresponding values in the “diabetes status model” as shown in Table 2.

**Table 3:** Adjusted odds ratios for thyroid cancer in diabetes duration model*.

Variables	Interpretation	Thyroid cancer case number	OR	95% CI	*P* value
Diabetes duration	<1 year vs. Non-diabetes	1/794	0.071	(0.010, 0.507)	0.0084
	1–3 years vs. Non-diabetes	12/794	0.450	(0.250, 0.813)	0.0081
	3–5 years vs. Non-diabetes	11/794	0.374	(0.203, 0.689)	0.0016
	≥5 years vs. Non-diabetes	125/794	1.159	(0.914, 1.470)	0.2224

* Other independent variables included in the model are the same as those shown in Table 2, except “diabetes status”. The OR (95% CI) for the other variables are not remarkably different from those shown in Table 2 and are not shown here. OR: odds ratio; CI: confidence interval.

## Discussion

In consistent with most previous studies [2], [3], thyroid cancer risk is significantly higher in women than in men (Table 2), supporting a potential role of estrogen in the growth and progression of thyroid cancer [14]. However, this study did not support an overall higher risk of thyroid cancer in patient with diabetes after multivariable adjustment (Table 2). Actually when categorized by diabetes duration, those with a diabetes duration of <5 years might show a significantly lower risk of thyroid cancer (“diabetes duration model” in Table 3). Although diabetic patients might have a higher probability of receiving examinations that might potentially lead to the diagnosis of thyroid cancer (Table 1), the findings could not be explained by such a detection bias (Tables 2 and 3).

A significantly lower risk of thyroid cancer in patients having diabetes diagnosed for <5 years was not previously reported. However, this was in line with the 12-year prospective follow-up study conducted in Europe showing an inverse association between blood glucose level and thyroid cancer risk [11]. Insulin resistance with elevated circulating insulin level is a cardinal feature of type 2 diabetes mellitus at the early stage, which has always been suggested as the etiology of diabetes-related cancer [6]. The observation of a lower risk of thyroid cancer in patients with diabetes duration <5 years (Table 3) may argue against such a hypothetical role of insulin resistance/hyperinsulinemia in the development of thyroid cancer, as advocated by some investigators [5]. However, other possibilities may also explain such a finding. For example, metformin may improve insulin resistance and reduce insulin level, counteracting the related mechanisms leading to thyroid proliferation or cancer [5]. Metformin may also reduce TSH level, decrease the size of benign thyroid nodules, suppresses the growth of thyroid carcinoma cells, and has multi-faceted properties protecting against cancer with the potential to be used for the treatment of thyroid cancer [5]. In patients with new-onset diabetes, metformin is very effective for glucose control and it is always recommended as the first-line therapy in these patients [15]. Therefore, it is possible that the widely used metformin in patients with early diabetes may protect against the development of thyroid cancer, as observed in the present study (Table 3). It is true that, though not statistically significant, metformin use was associated with a lower risk of thyroid cancer in the present study (Table 2). Although TSH level, a predictor for thyroid cancer [16], may be elevated in patients with type 2 diabetes in some studies [17], [18], this could not be confirmed in a recent study conducted in our Taiwanese population [19] or in studies from other ethnicities [20]. Furthermore, TSH did not correlate significantly with body mass index, fasting plasma glucose, hemoglobin A_1C_, insulin level or insulin resistance index in patients with diabetes in a Pakistani study [17]. It is not known whether the elevated TSH levels in the diabetic patients may only be observed in those with prolonged diabetes duration when sulfonylurea is added in the therapy regimen (to be discussed below).

The risk in patients with a diabetes duration ≥5 years became neutral (“diabetes duration model” in Table 3). One of the explanations is that with prolonged duration of diabetes, the interplay of the use of various anti-diabetic drugs and medications used for other comorbidities might have jointly influenced the risk of thyroid cancer in the diabetic patients. Addition of sulfonylurea is always considered when metformin alone can not satisfactorily lower blood glucose level in the diabetic patients [15]. Therefore, when diabetes progressed, the use of sulfonylurea might have increased the risk of thyroid cancer (Table 2, to be discussed below). But this would also be counteracted by other protective drugs such as statin or aspirin (Table 2, to be discussed below) for the treatment of dyslipidemia or cardiovascular disease, which are commonly seen in patients with a longer duration of diabetes.

First generation sulfonylureas have been well known to exert anti-thyroidal effects and may be goitrogenic in animals [21] or human beings [22]. These effects might lead to clinical or subclinical hypothyroidism with elevated TSH. It has been shown that a higher level of TSH, even within the normal range, may increase the risk of thyroid cancer [16]. Therefore, it is biologically plausible that thyroid cancer might be induced by prolonged use of sulfonylurea, resulting from a persistently higher level of TSH even within the normal range. Although such a goitrogenic effect is rarer in patients receiving second generation sulfonylurea, a more recent study still favored such a possibility [23].

Both insulin and sulfonylurea may increase insulin level, but only the use of sulfonylurea showed a significantly higher risk of thyroid cancer (Table 2). This could possibly be explained by the different effects of insulin on the thyroid gland. Insulin may augment thyroid hormone transcriptional action [24], and reduce TSH level probably through the effect of hypoglycemia on pituitary-thyroid secretory activity [25]. These are contradictory to the anti-thyroidal and TSH-elevating effects of sulfonylurea. The differential risk associated with sulfonylurea and insulin also suggested that hyperinsulinemia or insulin resistance alone might not be responsible for thyroid cell proliferation in patients with type 2 diabetes.

Many *in vitro* studies have suggested an inhibitory role of statin on the proliferation of thyroid cancer cell lines; and statins have been proposed as potential therapeutic agents for human thyroid cancer [26]. Similarly aspirin or non-steroidal anti-inflammatory drugs may exert anti-proliferative effects on thyroid cancer cells [27]. The results of the present study strongly supported these observations and suggested a potential role of statin and/or aspirin on the prevention of thyroid cancer. In patients with a longer duration of diabetes, dyslipidemia and cardiovascular disease may set in and the use of statin or aspirin may increase. Therefore, the interplay between the anti-diabetic drugs and the medications used for the treatment of comorbidites might have led to a neutral risk of thyroid cancer seen in patients with a diabetes duration ≥5 years (Table 3).

It is interesting to observe a lower risk of thyroid cancer associated with atherosclerotic disease or its associated risk factors such as hypertension, dyslipidemia, chronic obstructive pulmonary disease (a surrogate for smoking), and various macro- or microvascular diseases in this study (Table 2). Increased vascularity is an important feature of thyroid cancer [28], therefore it is not surprising to see such a negative association between thyroid cancer and atherosclerotic risk factors or vascular diseases. The negative association has not been reported previously and has an implication that vascular requirement might be important for the development of thyroid cancer. This observation may also explain the result of a pooled analysis of five prospective studies in the US showing a reduced risk of thyroid cancer among smokers with hazard ratio (95% confidence interval) of 0.68 (0.55–0.85) [29]. Another explanation for the lower risk of thyroid cancer associated with the vascular diseases is that these patients might have a higher probability of being treated with statin or aspirin, which have been shown to be preventive for thyroid cancer in previous studies [26], [27] and in the present study (Table 2). Although it is biologically plausible for a lower risk of thyroid cancer associated with these vascular diseases or their risk factors as discussed above, the accuracy of the diagnosis of these comorbidities by using the ICD-9-CM codes is not known and therefore the findings should better be reconfirmed by future studies.

Obesity has been shown to be an important risk factor for thyroid cancer in studies conducted in western countries [11]. However, contradictory finding of a negative association between body mass index and thyroid cancer risk was observed in a case-control study from China [12]. Another recent study also contrarily suggested that women with morbid obesity may have a lower prevalence of thyroid nodules [30]. Although the present study showed a lack of association between obesity diagnosis and thyroid cancer (Table 2), it should be recognized that in clinical practice, we do not usually label a patient with the diagnosis of obesity unless he or she is rather obese (probably when the body mass index is >30 kg/m^2^), when the use of some weight loosing agents are required and the labeling of an obesity diagnosis is necessary for reimbursement purpose. Furthermore, we did not have anthropometric data, such as body mass index or waist circumference, for analysis in this investigation. Therefore, future studies are required to explore the role of obesity in the development of thyroid cancer in our population. However, there remains a possibility that these obese patients might have been more vigorously treated with statin or aspirin for their higher risk of vascular complications; and these medications might have exerted some preventive effects on thyroid cancer.

The present study was in line with several previous studies showing benign thyroid disease as a significant risk factor for thyroid cancer [3]. Such an association either indicated a misdiagnosis of a malignant lesion as a benign thyroid condition, or that some benign thyroid disease may progress to malignancy.

Some studies suggested an association between high levels of socioeconomic status and thyroid cancer, but others found a negative association or a lack of association [3]. People living in Kao-Ping/Eastern regions had a significantly higher risk of thyroid cancer (Table 2), which might suggest some unknown geographically or socioeconomically related risk factors. Because ionizing radiation is a well recognized risk factor for thyroid cancer [3], it is not known whether inappropriately higher use of medically diagnostic or therapeutic radiation could be responsible. Some environmental chemicals may disrupt thyroid function and increase the risk of thyroid cancer, these include dioxins and polyhalogenated aromatic hydrocarbons [3]. Therefore, a serious and vigorous look for the possibility of a higher exposure to these environmental chemicals among residents in the Kao-Ping/Eastern regions of Taiwan should be warranted. Although nonionizing radiation such as the ultraviolet radiation may cause skin cancer, its link with other cancer such as thyroid cancer is less well documented. The Kao-Ping/Eastern regions in Taiwan are mainly under-developed and less urbanized. Most people living in these regions are farmers or fishermen, and they might have a higher chance of exposure to ultraviolet radiation from sunlight. The observation of a higher risk of thyroid cancer among these residents (Table 2) has paved a way to the investigation on the possible link between sunlight exposure and thyroid cancer.

Although some cases of thyroid cancer may have been misclassified, such an occurrence was probably low in the present study because labeled diagnoses should be printed on all prescriptions handed out to patients in Taiwan. Mislabeling of a cancer diagnosis would not be acceptable to patients when they saw the diagnosis. In secondary sensitivity analyses when the definition of thyroid cancer was restricted to the patients who had been issued a Severe Morbidity Card bearing a diagnosis of thyroid cancer, the results were similar and the conclusions remained unchanged (data not shown). The impact of a higher probability of receiving potential detection examinations in the diabetic patients was also minimal, because the odds ratios seen in Tables 2 and 3 would not remarkably change if the covariate of “potential detection examinations” was not entered into the models (data not shown).

This study has several strengths. It is population-based with a large nationally representative sample. The database included outpatients and inpatients and we caught the diagnoses from both sources. The use of medical record also reduced the potential bias related to self-reporting. We also excluded patients with type 1 diabetes to analyze the association specifically in patients with type 2 diabetes.

Limitations included a lack of actual measurement of anthropometric factors, smoking, alcohol drinking, family history, physical activity, lifestyle, diet, hormones and genetic parameters. In addition, we did not have biochemical data for evaluating their impact. Finally, it would be very important to know the histological feature of the thyroid cancer under study, but the present study was not able to evaluate the histological patterns, molecular markers or clinical stages because of lack of such information. However, according to the Taiwan Cancer Registry, papillary cancer represents 78.1% and 86.0% of all pathologically proven thyroid cancer in men and women, respectively [31], the findings of the present study might better be applied to thyroid papillary cancer.

In summary, this study shows a lack of an overall association between diabetes and thyroid cancer. Actually patients with diabetes duration <5 years may show a significantly reduced risk. With prolonged diabetes duration, a neutral association is observed. Hypertension, dyslipidemia, chronic obstructive pulmonary disease, and various macro- and microvascular diseases are consistently associated with a lower risk of thyroid cancer, indicating the importance of vascular requirement for the development of thyroid cancer. Statin, aspirin or non-steroidal anti-inflammatory drugs may be associated with a lower risk, but sulfonylurea may increase the risk of thyroid cancer.
